# Courtship, sex and poverty: illegitimacy in eighteenth-century Wales

**DOI:** 10.1080/03071022.2018.1394000

**Published:** 2017-12-19

**Authors:** Angela Joy Muir

**Affiliations:** ^a^ University of Exeter

**Keywords:** Illegitimacy, courtship, old poor law, Britain, Wales, eighteenth century, gender

## Abstract

This article sheds new light on illegitimacy in eighteenth-century Britain through an analysis of evidence from 36 parishes across the former Welsh counties Montgomeryshire and Radnorshire. Quantitative analysis of illegitimacy ratios demonstrates that levels were significantly higher in certain, but not all, parts of Wales in the eighteenth century. This evidence is considered in relation to explanatory frameworks used in the analysis of English data, which attempt to account for rising levels through cultural changes that influenced premarital sexual behaviour, and economic opportunities created by industrialization. Welsh evidence appears to present a challenge to these understandings in two key ways: Wales was linguistically different and lacked certain cultural markers which some historians have associated with an eighteenth-century ‘sexual revolution’, and because the highest levels of illegitimacy were found in agricultural regions of Wales which experienced little or no industrial change. It is argued that Welsh illegitimacy was influenced by a combination of courtship-led marriage customs, a decline in traditional forms of social control and worsening economic circumstances which, on closer examination, appear remarkably similar to London. This analysis provides further evidence that illegitimacy in eighteenth-century Britain was a deeply complex phenomenon governed by diverse regionally specific social and economic influences.

Most studies of illegitimacy in Britain have focused on English parishes, and a smaller, but no less important, body of scholarship investigating illegitimacy in eighteenth-century and nineteenth-century Scotland also exists.^1^ However, historians have tended to neglect Welsh material. This lacuna is surprising given both the availability and richness of Welsh sources, and the high levels of illegitimacy reported in certain parts of Wales in the nineteenth century. This article seeks to take some initial steps towards redressing this gap in the historiography of illegitimacy in Britain by presenting new quantitative data from 36 parishes across two Welsh counties. This evidence will then be considered in relation to explanatory frameworks used in the analysis of English illegitimacy, which attribute increasing levels of illegitimacy in the eighteenth century to changes in the types of sex unmarried people engaged in, and to changing economic circumstances. Although Welsh patterns of illegitimacy did roughly resemble trends elsewhere in Britain, they differed in significant ways. Central to understanding these patterns are the cultural and economic contexts within which these changes occurred.

The birth of a child outside of wedlock was a familiar occurrence in eighteenth-century Britain. Extensive demographic studies, such as those of Peter Laslett in the 1970s and subsequent works such as that of Richard Adair in the 1990s, have been invaluable in revealing just how common these experiences were in some areas of England.^2^ These studies uncovered broad national trends, with levels increasing from approximately 1.7% of all births born outside of wedlock in the late seventeenth century, to approximately 3% of all births by 1750, and nearly doubling again by the start of the nineteenth century.^3^ These studies have also highlighted considerable regional variations and local persistence, with urban parishes, and parishes in the north and west of England consistently experiencing higher levels of illegitimacy than rural parishes, and areas in the south and east. Moreover, the parishes which reported high illegitimacy for one period typically continued to report levels consistently above the national average over subsequent decades.^4^ The circumstances which influenced these patterns of continuity and change have been a source of much debate over the past four decades, with historians revisiting the question from diverse social, economic and cultural perspectives.

One of the biggest questions historians of illegitimacy have tried to answer is why so many unmarried women bore children outside wedlock. Hypotheses have included Laslett’s ‘bastardy prone sub-society’ and theories which relate either to non-marital cohabitation and irregular marriage, to changes in sexual practice resulting from shifting attitudes towards penetrative sex, and to conjugal courtship customs whereby intended marriages were either delayed or thwarted as a result of changing socioeconomic circumstances.^5^ For the purposes of this article, Welsh evidence will be considered in relation to theories about changes in sexual practice, and to theories about socioeconomic influences on courtship. Historians such as Tim Hitchcock, Thomas Laqueur, Randolph Trumbach and Faramerz Dabhoiwala have argued that the types of heterosexual sexual activity men and women engaged in changed during the eighteenth century, displacing activities such as mutual masturbation with penetrative sex, which resulted in more children being conceived and born outside of marriage.^6^ Other historians, such as Peter Laslett, Richard Adair, Thomas Nutt and Nicholas Rogers have utilized various demographic, social and economic approaches to parish and court records to account for rising levels of illegitimacy.^7^ Central to these approaches is the understanding that most illegitimacy resulted from courtships in which couples engaged in penetrative sex with the intention (or at least promise) of marriage, but failed to marry when pregnancy occurred.^8^ Such failures are often attributed to economic circumstances which prevented couples from marrying, and thus the influence of industrialization in the eighteenth century is often central to these discussions.^9^

These two broad explanatory frameworks are not entirely at odds with each other, and do help shed light on different aspects of the tremendously complex phenomenon of plebeian sexual culture in the eighteenth century, which is notoriously difficult to access. It is therefore useful to consider these in tandem.^10^ The extensive work which has been done within these broad approaches is compelling and convincing, and contributes a great deal to our understanding of illegitimacy in eighteenth-century England. Historians such as Emma Griffin and Adrian Wilson have integrated elements of these two broad approaches to explore the ways in which sexual practice intersected with changing economic circumstances to produce a larger number of illegitimate children in the eighteenth century.^11^ These integrated approaches provide a useful starting point for analysis of Welsh illegitimacy, and also allow for the Welsh context to be drawn into the debates about illegitimacy in Britain more broadly. Levels of illegitimacy will be considered first, and will be followed by analysis of sexual culture and courtship in Wales, and an examination of economic circumstances within which these activities took place. What this analysis reveals is the extent to which illegitimacy levels were acutely sensitive to economic hardship, the effects of which were amplified when particular types of courtship practices were present.

## Illegitimacy in Wales

Research on illegitimacy in Wales has been limited to three postgraduate studies and one published article, the latter of which consists of a limited quantitative study of two parishes undertaken by this author.^12^ The omission of Welsh data from larger studies of English data is surprising given the availability of source material for most regions, and given that the 1836 *Annual Report of the Poor Law Commission* revealed that the counties of Radnorshire and Montgomeryshire contained the greatest proportion of ‘chargeable bastards’ per capita in all of Britain, with rates of 1:59 and 1:67 respectively.^13^ From the 1530s onwards England and Wales shared a similar secular and ecclesiastical legal system, which means demographic data from Welsh parishes can be collected and analysed using similar methodologies as those used in the analysis of English parish data, thus allowing for direct comparison. For the present study, the illegitimacy ratio has been calculated per 10-year period for 36 parishes in the counties of Montgomeryshire and Radnorshire between the years 1680 and 1799. Both counties shared borders with England, and in the case of Montgomeryshire, extended as far west as the Dyfi estuary, which opens into the Irish Sea. The data represents 81,573 baptisms across the period, increasing from just below 4,000 in the first decade of the study to just over 9,700 in the last.^14^ Calculating levels of illegitimacy during the parish registration era is done by dividing the number of illegitimate children recorded in a baptism register over a 5 or 10-year period by the total number of baptisms over the same period.^15^ This is an illegitimacy ratio rather than an illegitimacy rate, as the latter refers to the number per 1,000 unmarried women of child bearing age. When twins or triplets are identified, they are counted as one entry as they represent one conception. Older children and adults, whether legitimate or illegitimate, are not counted. Only data collected from baptism registers has been included in the ratios. Unbaptized illegitimate children can often appear in other records such as poor law accounts and burial registers; however, these children have not been counted.^16^ The reasons for this are twofold: first, the ages of children are frequently not given in burial registers so it is not always possible to know in which birth year to include an illegitimate burial. Second, and most importantly, including illegitimate infants found in burial registers risks inflating the illegitimate ratio because it is not always possible to identify and include unbaptized legitimate burials. Since no adjustment can be made to include both legitimate and illegitimate burials, no burials have been included in the ratios. It should be noted that many of the family reconstitution techniques used by historical demographers for the study of illegitimacy and family structure, such as calculating prenuptial pregnancy, and identifying ‘repeaters’, or women who bore more than one illegitimate child, cannot be applied to Welsh sources. This is due to the limited pool of Welsh surnames which makes it difficult or impossible to differentiate between different individuals who carry the same names.^17^

**Figure 1.**
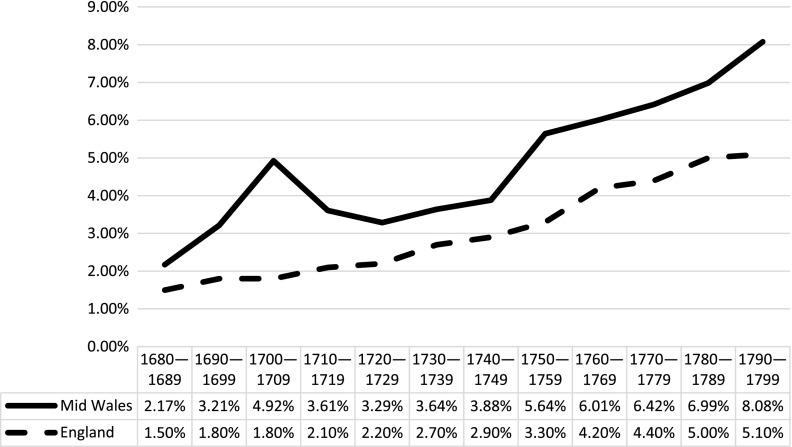
Comparative illegitimacy ratios in mid Wales and England, 1680–1799. Sources: Welsh data aggregate values of sources listed in note 14; English data from Laslett, ‘Introduction’, *Bastardy, op. cit.*, 14.

Figure 1 compares the aggregate findings from 36 Welsh parishes to Laslett’s 98 English parishes.^18^ Levels of reported illegitimacy in mid-Wales throughout the late seventeenth and eighteenth centuries were consistently higher – and at times significantly so – than the overall average for England. The illegitimacy ratio in mid-Wales rose from just below 3 to 8% over the 120 year period, with one early peak of 5% in the decade 1700 to 1709. Other than the early peak, the trend in Wales appears to have followed roughly the same pattern as in England, but with increasing divergence over the course of the period. In the 1680s the difference between mid-Wales and England was approximately 1.2%, whereas by the end of the eighteenth century it had increased to nearly 3%. Although levels of illegitimacy in England were also on the rise during this period, overall levels in Wales were consistently higher, and the marked differences noted by poor law reports in the 1830s were evident in some parts of Wales at least 100 years earlier. Some of the factors which may have contributed to both the increase and higher levels overall will be explored more below.

**Figure 2.**
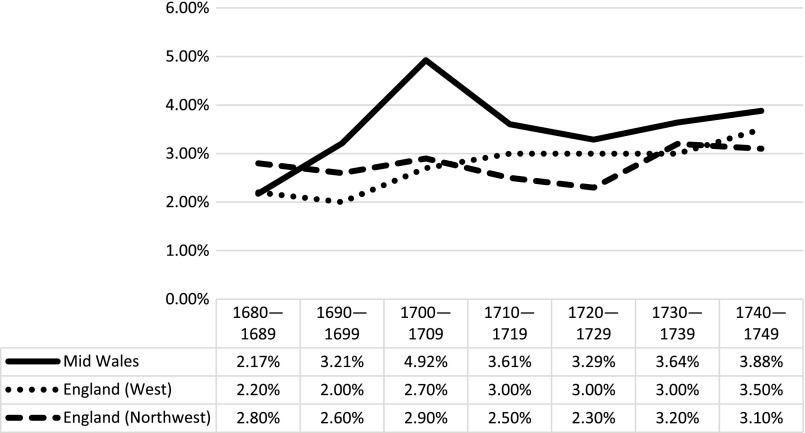
Comparative illegitimacy ratios in mid Wales and English regions, 1680–1749. Sources: see note 14; English data from Adair, *op. cit.*, 51–62.

In his study of 250 English parishes between the 1530s and 1750s, Adair distinguished between highland regions in the north and west, and lowland regions in the south and east of England, and found that highland regions consistently experienced higher levels of illegitimacy throughout the period.^19^ Most of the Welsh parishes considered here are highland, other than a handful of parishes in the lowlands of the Severn valley in eastern Montgomeryshire, so a highland/lowland distinction is not appropriate here. However, regional variations which may not be linked to topography are still evident. Comparisons can be made with Adair’s regional data for the latter part of the seventeenth and early eighteenth centuries. The most relevant regions for comparison are Adair’s west and northwest regions, as the western region includes counties bordering Montgomeryshire and Radnorshire, and the northwest region shows a roughly analogous pattern of change over time (see Figure 2).^20^ Although illegitimacy ratios in mid-Wales were constantly higher than in western England, the upward trend across both regions from the 1720s is notably similar. All three regions began with a ratio of between 2 and 3% in the 1680s, and ended with between 3 and 4% in the 1740s, which was the last complete decade studied by Adair.

**Figure 3.**
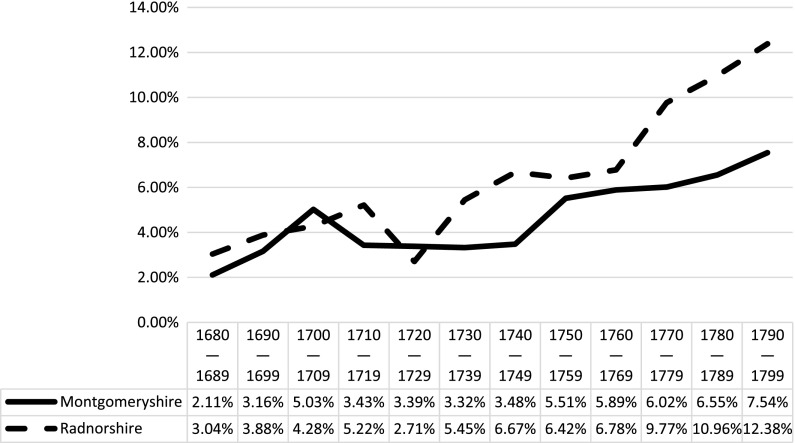
County-level analysis of illegitimacy ratios in Montgomeryshire and Radnorshire, 1680–1799. Sources: see note 14.

**Figure 4.**
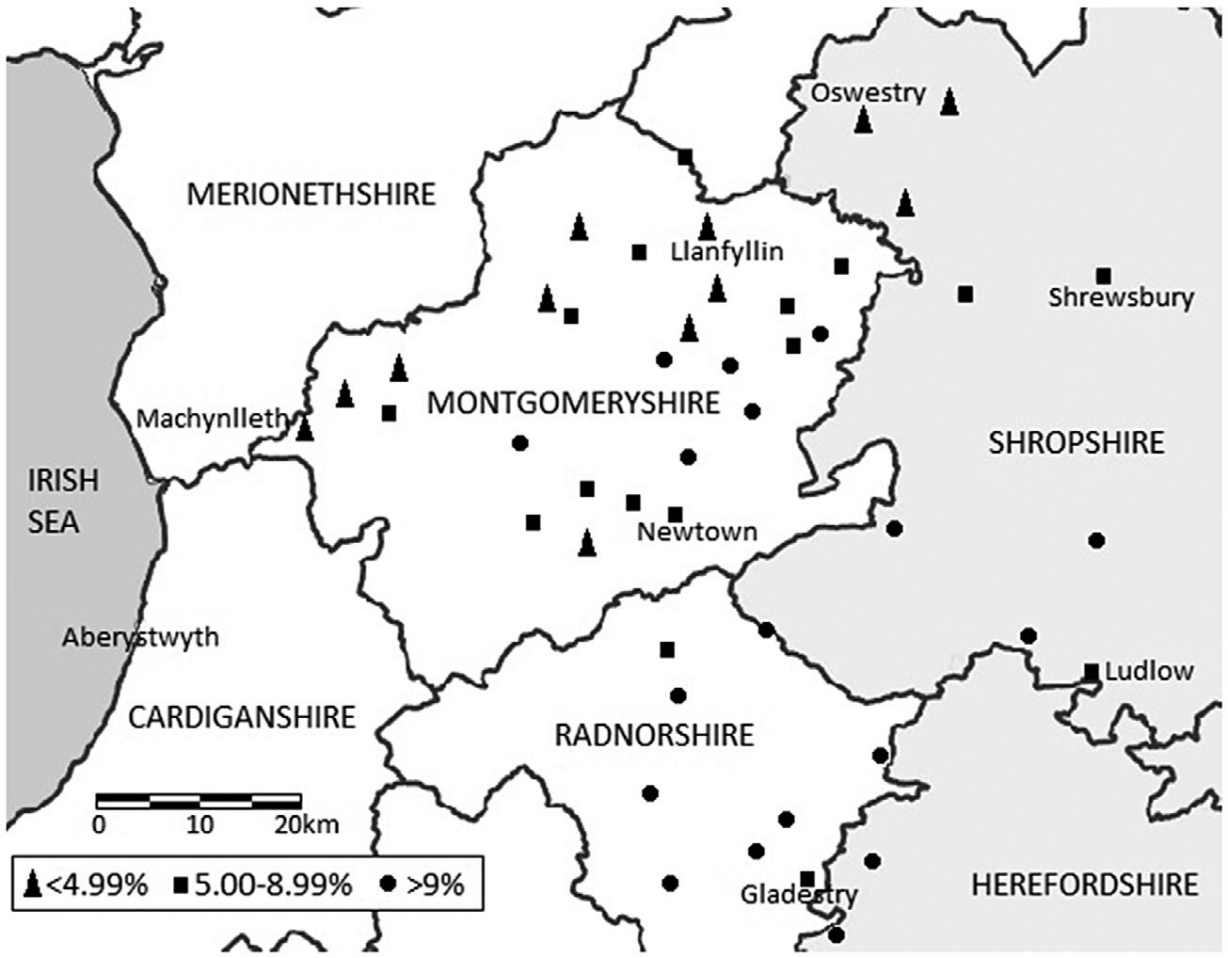
Map of illegitimacy ratios by parish, 1790–1799: Montgomeryshire, Radnorshire, Shropshire and Herefordshire. Sources: see notes 14 and 21.

County-level analysis within the study region reveals even more regional variation, and suggests that there may not have been a singular ‘Welsh’ experience of bearing a child outside of wedlock. As Figure 3 demonstrates, levels of reported illegitimacy in Radnorshire were considerably higher than in Montgomeryshire, and became increasingly so towards the end of the eighteenth century. The increase in ratio in Radnorshire in particular is striking, and suggests significant continuity with the levels reported in the 1830s. Considerable variation is also apparent within each county. As Figure 4 demonstrates, the parishes which experienced the highest levels of illegitimacy are in the east and are closest to the border regions with England.^21^ The parishes with the lowest levels of illegitimacy are located much further west in predominantly pastoral upland regions, some of which were involved in the woollen trade.^22^ Moreover, in the last decade of the eighteenth century many lowland parishes located within the boundaries of the English counties of Herefordshire and Shropshire had illegitimacy ratios which exceeded the ratios of some Welsh parishes. This strongly suggests that the factors which contributed to increased levels of illegitimacy were tied to more diverse socioeconomic regional variations rather than distinctive ‘national’ characteristics. These local and regional variations, which are frequently masked by broader statistical trends, are significant and warrant further investigation. In understanding the diversity of reproductive experiences in the eighteenth century the outliers are arguably as important as the average.

**Figure 5.**
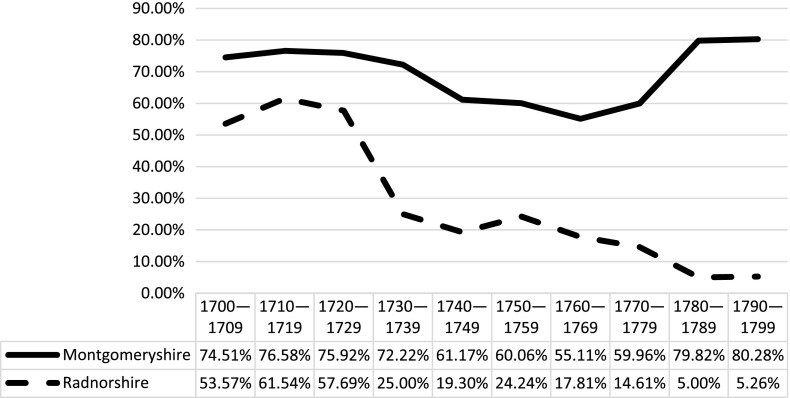
Percentage of baptisms of illegitimate children in which fathers were named. Sources: see note 14.

Another compelling pattern which is apparent in many Welsh baptism registers is the frequency with which unmarried fathers were identified at the time of baptism. In many parts of England, unmarried fathers were identifiable in fewer than 50% of cases and in some areas in as few as 7% of cases.^23^ However, some Welsh parishes identified unmarried fathers in over 80% of cases. This phenomenon is significant because it provides clues about the socioeconomic background of fathers, and the relationships between the unmarried men and women who begat and bore children in the eighteenth century. As with patterns of illegitimacy, the evidence of identified illegitimate paternity reveals marked regional variation, and both continuity and change over time. As Figure 5 demonstrates, fathers in Montgomeryshire were consistently named in between 55 and 80% of cases, whereas in Radnorshire identification decreased from more than half of unmarried fathers identified at the start of the century, to almost none being named at the end. When considered in relation to broader cultural and economic circumstances, this phenomenon may be central to our understanding of the causes and nature of illegitimacy in eighteenth-century Wales.

Taking into account the exceptionally high levels of illegitimacy seen in mid-Wales, and Radnorshire in particular, which had levels more than double the national average in England, it is clear that something changed which either enabled or encouraged Welsh men and women to have more procreative sex outside of marriage. Instances of recorded illegitimacy do not represent every instance of non-marital sex which took place, and therefore baptism registers underrepresent the amount of sex unmarried men and women were having.^24^ For an illegitimate child to be recorded in parish registers a series of events must have occurred.^25^ A man and a woman who were not formally married had to engage in sexual intercourse, resulting in the conception of a child; the monthly probability of conception occurring for women engaging in regular sexual intercourse has been estimated as between 15 and 50%.^26^ Prior to birth the mother must not have married any man, as that would have legitimized the child. The pregnancy would have to go as near to full term as necessary for a viable live birth without having been terminated by a natural miscarriage or an induced abortion. The probability of a fertilized ovum naturally surviving to nine months was roughly 50%.^27^ The infant would then have to survive the precarious first few hours and days of life when it was most at risk of succumbing to death by natural causes, which typically claimed between 110 and 170 new born lives per 1,000 births.^28^ This does not take into account the small fraction of children who were victims of abandonment or infanticide. A parent or guardian, such as a grandparent, would then have to bring the child to their parish church for baptism. Finally, parish officials must have had enough information about the parent or parents to be able to identify the child as illegitimate, and feel compelled to record the child as such. When this list of requisite circumstances is considered alongside an illegitimacy ratio of between 7 and 12% it becomes apparent that a sizeable proportion of unmarried men and women in Wales were engaging in considerably more pre- or extra-marital sex by the end of the eighteenth century than their counterparts a century earlier. Such a significant shift was unlikely to have been caused by a single phenomenon, but rather by a confluence of influences.

## A Welsh sexual revolution?

Many historians maintain that courtship was the leading cause of childbirth outside of marriage in eighteenth-century Britain, and Richard Adair has suggested that high levels of identified illegitimate paternity may be indicative of conjugal courtship customs.^29^ Any unmarried woman of reproductive age was at risk of bearing an illegitimate child.^30^ That risk increased substantially if she was engaging in regular sexual contact, even if penetrative sex only took place during the latter, most serious stages of courtship, because interruption in the marriage process could have resulted in a child being born outside of wedlock. Unfortunately, the vast majority of men and women involved in illegitimacy in the eighteenth century left no accounts of their own experiences. It is clear that not all illegitimate children were conceived under similar circumstances, which makes accounting for broad trends and change over time problematic. Ample evidence of exploitative sexual relationships exists, and it seems deeply inappropriate to count children born as a result of rape or incest in the same category of sexual experience as consensual premarital sex.^31^ Moreover, it is likely that many unmarried men and women engaged in consensual sexual activity with no intention of marrying at all, as can be seen in cases of adultery or prostitution. With the exception of only the most detailed entries, parish registers unfortunately provide no way for modern researchers to distinguish between different types of consensual and non-consensual procreative sex. Therefore, it is important to bear in mind that considering illegitimacy ratios in aggregate obscures the diversity of lived sexual experiences, and that there can be no universal explanation for why children were conceived outside of wedlock. Nevertheless, it is highly probable that many, if not most, illegitimate children born in eighteenth-century Britain were conceived with the intention to marry on the part of one or both parties.

Emma Griffin’s analysis of plebeian autobiographies found that unmarried men and women did sometimes engage in sexual activity either on promise of marriage, or in hopes of securing a marriage.^32^ Unfortunately, similar first-hand accounts do not exist for Wales. However, certain Court of Great Sessions records do indicate that sex was very much a part of pre-marital courtship for some couples, at least from 1750 onwards. The most compelling evidence comes from murder trials.^33^ Between 1750 and 1800 five pregnant single women were murdered, and in each of the cases the accused was the supposed father of the child, and the relationship between victim and accused was understood to be one of courtship.^34^ In the 1764 murder of Elizabeth Evans by Evan Jenkins, Jenkins’s employer deposed that he believed the two were intending to marry as the banns had been published.^35^ In the 1788 murder of Margaret Thomas, a witness deposed that she had heard the accused, William Williams, asking Margaret to publish banns so the two could marry.^36^ In 1756, Margaret Matthews was murdered, and the lone suspect was Edward Pugh, who witnesses said was courting her. One witnessed deposed that on the night she disappeared, Margaret went searching for Edward, ‘to know his answer… whether he would have her or take care of her and her child’.^37^ Caution should be taken when considering these cases in terms of the broader experience of courtship in Wales as they are in many ways exceptional. These women were all murdered by men whom they had hoped to marry, and those sentiments were clearly not reciprocated. For example, Evan Jenkins confessed to his gaoler that he ‘would rot in iron before he would marry [Elizabeth]’.^38^ However, it is entirely plausible that the victims and their communities genuinely believed these relationships would eventually be solemnized, and perhaps these women had hoped that by becoming pregnant their partners would be compelled to marry them.^39^ Although both tragic and exceptional, these examples do demonstrate that pre-marital penetrative sex was taking place in Wales within the context of courtship, and communities were aware of these relationships.

Welsh courtship customs which could have involved sexual contact include practices such as bundling, or *caru ar* [or *yn*] *y gwely*, or ‘love on[or in] the bed’.^40^ Bundling entailed a young couple spending an evening, and frequently an entire night, together, unsupervised, in bed, typically in the home of the young woman.^41^ If and when the woman conceived, marriage was the expected outcome. The origins of the custom are not clear, but it has been argued that it was either a form of fertility testing or trial marriage to test a couple’s compatibility.^42^ Although not unique to Wales, it is the courtship custom most frequently associated with the country.^43^ Evidence of the practice and what it involved is scarce. As a popular custom of the rural poor no first-hand written accounts of bundling in the eighteenth century exist, but reference to it can be found in some official nineteenth-century accounts. Evidence from the eighteenth century is scarce, but the accounts that do exist are richer in detail. Intriguingly, some English travel writers who claimed to have witnessed the custom depicted it as a polite one which did not involve any sexual contact at all. An English traveller described the bundling he witnessed while staying as a guest in a Welsh home in Merionethshire in 1772 as completely modest, contrary to accounts of Welsh virtue he had previously heard.^44^ Another particularly detailed account described bundling as an innocent ritual, while also providing further insight into the socioeconomic background of those who participated in it:

The lower order of people do actually carry on their love affairs in bed, and what would extremely astonish more polished lovers, they are carried on honourably, it being, at least, as usual … to go from the bed of courtship to the bed of marriage, as unpolluted and maidenly … and yet, you are not to conclude that this proceeds from their being less susceptible of the belle passion than their betters.^45^

These accounts are recorded by English visitors travelling through Wales commenting on the antiquities and novelties of ‘Welshness’. It is difficult to know if the accounts of outside observers represent an ‘authentic’ description of customs which they may not have fully understood, or to which they may not have been completely privy. However, these conflicting descriptions may also be indicative of changes in sexual practice that took place during the century and which may account for increasing levels of illegitimacy.

The authors of the 1834 Rural and Town Queries, which heavily influenced subsequent poor law reform, identified bundling as a ‘powerful cause’ behind the high levels of illegitimacy reported in nineteenth-century Wales.^46^ The report described bundling as ‘the way in which all courtships amongst the lower class have been carried on from time immemorial’, and it was not considered ‘either immoral or indecent’.^47^ Other than referring to bundling as ‘courting in bed’, the report offered no description of what courting couples actually did, although we can implicitly assume the authors believed some were engaging in penetrative sex, as the practice often resulted in pregnancy. Fragments of evidence can also be found in court records from the nineteenth century. In Carmarthenshire in 1816 a yeoman and lay preacher named Rees Thomas Rees from Llangadog was convicted of murdering Elizabeth Jones, his 19-year-old lover. Elizabeth’s father testified that Rees had been ‘in the habit of sitting up with the deceased in [her] house for the purpose of courting her as was customary in the county’.^48^ Elizabeth became pregnant, and Rees convinced her to consume a mixture of arsenic and mercury in an attempt to induce an abortion; Elizabeth died as a result. Although not referred to specifically by name as bundling or *caru ar y gwely*, it is likely to have been the same practice mentioned by the authors of Rural and Town Queries. Eight years later in Anglesey, a labourer named Griffith Roberts was charged with burglary for unlawfully entering a dwelling.^49^ His defence was that he had entered the property with the intention of courting a servant in the household, and he was never indicted. Such a defence would only be possible if it was customary for young men to secretly enter the homes of the women they were courting at night. It is highly plausible, therefore, that bundling was part of plebeian courtship in some parts of Wales, at least in the nineteenth century. In certain circumstances, it clearly did create an opportunity for couples to engage in penetrative sex, but how many actually did so is impossible to ascertain.

Much has been written about heterosexual sex in the seventeenth and eighteenth centuries.^50^ Some historians have argued that the type of sex men and women were engaging in changed, and such changes were part of a broader sexual revolution which was brought on by the economic growth and social change resulting from increasing industrialization, the Enlightenment, and the decline in ecclesiastical regulation of sexual behaviour.^51^ As a result, perceptions about male and female sexuality shifted between in the late seventeenth and early eighteenth centuries, whereby women went from being understood in cultural, medical and legal terms as sexually aggressive and lustful to being perceived as sexually passive.^52^ This was mirrored by a correlative emergence of a new active male sexuality, which emphasized penetrative sex. Tim Hitchcock describes this as a shift away from non-penetrative sexual activity, such as mutual masturbation, towards more ‘phallo-centric’ sex.^53^ Such a shift would have been particularly significant for unmarried couples engaging in sexual activity, as more penetrative sex outside of marriage would have resulted in more childbirths outside of marriage. This change in sexual practices is used to account for, at least in part, the increasing levels of illegitimacy seen in the eighteenth century. Hitchchock maps these changes onto the growing popularity of proscriptive literature which increasingly denounced the sin of Onan, associated particularly, but not only, with male masturbation. The earliest English-language example of this type of literature was *Rebuke of the Sin of Uncleanness* by Josiah Woodward, which was published by the Society for the Promotion of Christian Knowledge (SPCK).^54^ Later and more popular examples include the anonymous *Onania, or the Henious Sin of Self*-*Pollution, and all its Frightful Consequences in both Sexes…*, which was published in 1708, and Tissot’s *Onanism: or a treatise upon the dissorders produced by masturbation*, which was published in French in 1758 and subsequently translated into English.^55^ Other advice literature, such as *Aristotle’s Master*-*piece: or, the secrets of generation displayed in all the parts thereof*, which was first published in 1684*,* and Nicholas Venette’s *Tableau de l’amour conjugal*, first translated into English in 1703, were manuals of reproductive knowledge. These works were not so much anti-masturbatory as they were ‘pro-natalist’.^56^ *Onanism* and *Aristotle’s Masterpiece* remained in publication until the twentieth century.^57^ The significance of this type of literature was that it propagated a type of sexual knowledge which emphasized the importance of the penis in reproduction, and resulted in a profound shift in sexual practice.^58^ How far these ideas disseminated throughout society is impossible to measure, but the persistent and increasing popularity of the text would indicate a wide reception.

The presence of similar literature in Wales may therefore be an indicator of whether Wales experienced a similar revolution in sexual practice. Rising levels of illegitimacy, and conflicting descriptions of bundling may suggest that sexual culture changed during the eighteenth century, particularly for courting couples. It is possible that descriptions such as Mr Pratt’s reflect earlier non-penetrative manifestations of bundling which, by the nineteenth century, had evolved to include penetrative sex. However, the types of evidence Hitchcock uses in his analysis of sexual change in England is conspicuously absent in Wales. Before 1800, Wales remained predominantly monoglot Welsh, although areas such as Pembrokeshire and communities closer to the English border, and in industrializing regions of the south, would have had larger English-speaking populations, and many would have been bilingual.^59^ The existence of these texts in Welsh would therefore provide persuasive evidence of a similar shift in Wales. An increasing number of printing presses dedicated to the printing of Welsh-language materials were founded from the early 1700s, and by 1800 virtually every Welsh town had at least one press.^60^ Despite there being the means to reproduce such texts, the vast majority of works published by these presses were of a religious nature.^61^ Crucially, not a single Welsh translation of any of these works was produced during the eighteenth century, and there was no original Welsh-language equivalent.^62^ *Aristotle’s Masterpiece* was translated into Welsh as *Gwaith Aristotle*, but not until 1826. The SPCK, which was active in Wales in the eighteenth century, did translate some of Josiah Woodward’s works into Welsh, however *Rebuke of the Sin of Uncleanness* does not appear to be one of them.^63^ There is therefore no clear link between the ideas communicated in these genres of literature and Welsh sexual culture in the eighteenth century. It is possible that ideas about anti-masturbatory, pro-penetrative sex were disseminated throughout Wales via oral culture. Research into medical knowledge in eighteenth-century Wales has provided convincing evidence that information about medicine and remedies found in popular medical texts were translated and passed through oral communication, and then transcribed in personal diaries and household accounts.^64^ However, given the complete absence of any proscriptive literature about sexual conduct in the Welsh language in the eighteenth century, its impact on the types of sex unmarried Welsh men and women were having should not be overstated.

Despite the absence of pro-penetrative Welsh literature, it is still possible that sexual culture in Wales changed during the eighteenth century, but for entirely different reasons. Moreover, given that illegitimacy levels in Wales increased without a clear correlation with the types of literature examined by Hitchcock, it may be that these texts were not as instrumental as he suggests.^65^ Alternatively, Faramerz Dabhoiwala has identified the decline in legal regulation of sex outside of marriage as one of the central features of what he sees as an eighteenth-century sexual revolution.^66^ To suggest the changes which occurred in Wales amounted to a sexual ‘revolution’ would be an overstatement, but a connection with the decline in ecclesiastical regulation can still be made. Throughout the early modern period, English and Welsh plebeian sexual activity had been regulated by the church, and sexual transgressions were dealt with by church courts.^67^ However, from the late seventeenth century onwards the authority of church courts began to wane, and by the late eighteenth century local ecclesiastical authorities were increasingly reluctant to prosecute for sexual transgressions such as premarital fornication and bastardy.^68^ Subsequently, levels of reported illegitimacy in some areas began to rise.^69^ Throughout the eighteenth century moral reformers did make repeated attempts to legislate against certain types of illicit sexual activity, but by the middle of the eighteenth century most consensual sex outside of marriage was no longer the concern of the courts.^70^ Furthermore, the religious changes associated with the rise of nonconformity in Wales would have had little influence on plebeian sexuality in eighteenth-century mid-Wales. The vacuum created by the decline in the authority of ecclesiastical courts was not immediately filled by the moral strictures of Methodism. Although Wales in the nineteenth century is synonymous with staunch religious nonconformity, it is easy to exaggerate its impact prior to 1780.^71^ The rise of Methodism was slow and fitful, and its message appealed more to the middling sort, particularly in towns, than to the poor labourers and paupers who constituted the bulk of the population. The circulating schools of Griffith Jones, which led to substantial increases in literacy and Nonconformity, were not overly active in the area covered by this study.^72^ Nonconformity did increase in the latter decades of the eighteenth century, and chapels began to be established in Montgomeryshire as early as the 1790s, but their numbers would not have been statistically significant enough to influence plebeian sexual behaviour more widely, especially earlier in the century.^73^

It is likely that the decline in formal moral regulation of sexual behaviour did result in increased sexual freedom for courting couples, and perhaps even accelerated the speed with which some couples progressed to penetrative sex.^74^ The existence of customs which already allowed young couples a degree of freedom and privacy during courtship may well have accentuated the impact of this decline. However, the waning of ecclesiastical authority over pre- and extra-marital sexual behaviour on its own cannot entirely explain the changes in illegitimacy levels observed in Wales, as a similar decline occurred across England and Wales. Moreover, families and neighbours would still have viewed illicit sexual activity as a cause for concern. Communities would have played an active role in monitoring the behaviour of young, unmarried men and women, as few would have welcomed an increase in illegitimate births.^75^ The steps taken by poor law officials and secular courts to regulate and manage illegitimacy demonstrate the extent to which illegitimacy was seen as a significant problem throughout the eighteenth century. However, their concerns were practical rather than moral, as children born outside of wedlock posed a financial risk to communities.^76^ Sex within courtship may have been more acceptable to communities than other types of non-marital sexual relationships, but only if it resulted in marriage.^77^ However, a significant proportion of children conceived under these circumstances in Wales were not born to married parents, which raises the question of why so many Welsh couples were engaging in courtship practices but never marrying.

## The economics of marriage and illegitimacy

The most widely explored explanations for why courting couples failed to marry are related to economic circumstances. Peter Laslett codified one version of this in his ‘courtship intensity’ hypothesis, which assumes that couples based their courtship decisions on their financial prospects and ability to establish their own home and independence. This hypothesis, which also correlates with changes in the age of first marriage, posits that during periods of perceived economic stability marriage prospects for young people would appear good, therefore the age of marriage would drop, and more couples would engage in intensive courtship which involved penetrative sex. Increased sexual activity resulted in increased prenuptial pregnancy when the courtship was successful, and increased illegitimacy when the courtship failed or was interrupted. A significant proportion of illegitimate births were therefore ‘accidents of courtship’ which resulted from intensified courtship activity under more prosperous economic circumstances.^78^ Conversely, when economic prospects seemed poor, young people refrained from intensive courtship practices. Therefore, higher levels of illegitimacy should correlate with improved economic circumstances. Not all historians are convinced by the courtship intensity hypothesis. David Levine has argued that although economic changes may have delayed the age at which couples married, economic factors may not have influenced sexual behaviour outside of marriage.^79^ Emma Griffin has argued that this broad theory cannot account for the diverse variations found in regional studies, and furthermore, it is impossible to know how many couples within a community were actively engaging in courtship at any given time.^80^ Adrian Wilson has found more convincing evidence of a correlation between precarious economic circumstances – as evidenced by increasing grain prices resulting from bad harvest – and higher levels of illegitimacy in London, which were manifested in the number of women seeking reprieve from the Foundling Hospital.^81^ Wilson argues that these circumstances were compounded by courtship practices which differed to those found in rural England. This model may work in relation to Welsh evidence, and will be explored further below.

Economic explanations for increases in illegitimacy have also considered the influence of industrialization on courtship and marriage patterns. Edward Shorter was the first to explore this correlation by suggesting that industrialization resulted in higher illegitimacy by granting women economic independence, and therefore greater sexual freedom.^82^ The central assumption of Shorter’s argument, the notion that increased illegitimacy resulted from female emancipation, was quickly rejected by historians.^83^ In their response to Shorter, Tilly *et al.* argued that increasing levels of illegitimacy actually resulted from ‘structural and compositional changes associated with urbanization and industrialization’.^84^ According to them, illegitimacy was a by-product of the geographic mobility caused by industrialization and urbanization, which led to more women moving away from the support of their families, and more men facing economic instability resulting in more broken marriage promises.^85^ Nigel Goose has argued that slightly higher levels of illegitimacy in counties where women were employed in the straw plait trade could plausibly be connected to the effects of the trade on female employment, but also suggested this correlation should not be overstated.^86^ Emma Griffin also refutes Shorter’s claim of eighteenth-century female sexual liberation; however, her study of plebeian autobiographies does reveal an increase in sexual freedom for young, unmarried men and women. She also found that female employment opportunities created by industrialization could grant some women greater economic freedom, which enabled some to support children outside of marriage.^87^

These analyses are useful for understanding illegitimacy within the context of urbanization and industrialization, but do little to further our understanding of illegitimacy in rural contexts. Many studies have found that levels of illegitimacy in the nineteenth century were lowest in rural areas where traditional forms of domestic and agrarian employment persisted, and where long-established networks of family and community surveillance existed.^88^ However, mid-Wales had some of the highest levels of illegitimacy in the eighteenth and nineteenth centuries, yet remained almost exclusively rural and sparsely populated. Clearly, economic circumstances in rural, agrarian areas were different to those in urban and industrializing areas, but this does not preclude the application of certain parts of these approaches to a rural context. As has been repeatedly found, illegitimacy is a compound phenomenon which manifests differently in different regions, and therefore regional responses to changing socioeconomic variables would also differ.^89^ Given the nature of the available evidence, the most useful approaches for comparison in this preliminary analysis of Welsh evidence are ones which consider changes in courtship practice within the context of the socioeconomic climate, as both Griffin and Wilson have adopted for urban and industrializing regions. Having already explored changes in premarital sexual practice, I will now, therefore, turn to the economic circumstances of mid-Wales during the eighteenth century.

Montgomeryshire was the third largest county in Wales in the eighteenth century in terms of size, but in terms of population it ranked sixth, with a population which grew from approximately 31,000 in 1680 to 51,000 by the time of the first census in 1801.^90^ Radnorshire was the least populated county with a population in 1801 of approximately 19,000, and was one of only two Welsh counties with a population of less than 30,000.^91^ Despite the growth in population, the vast majority of people in Wales lived in sparsely populated rural areas. At the beginning of the eighteenth century the largest town in Wales was Wrexham, which had an estimated population of between 3,000 and 5,000, followed by smaller market towns such as Carmarthen and Brecon with populations nearer 1,000.^92^ The largest communities in Montgomeryshire included Newtown and Llandeilo Fawr, and in Radnorshire included towns such as Knighton, which could be described as ‘little more than villages’ with populations that still fell short of 1,000 by 1801.^93^ By the end of the century larger urban centres were beginning to develop as a result of industry in south Wales. However, the parishes covered by the current study had not experienced similar urbanization.

Many an English traveller commented on the Welsh landscape, which they saw as bleak and wild. The harshest comment described Wales as ‘the fag end of creation; the very rubbish of Noah’s flood’.^94^ Such observations reflected the reality that the terrain in many parts of the country offered scarcely more than meagre subsistence. The regions in question ranged between upland moors to the west, which were best suited for sheep grazing, and arable lowlands to the east, which produced crops such as oats and root vegetables which supported both the modest human diet and livestock, especially during the lean winter months.^95^ Agricultural advances which were seen across England during the eighteenth century had little impact on impoverished and remote parts of Wales and life for many was lived precariously from season to season at the mercy of each harvest.^96^ The nature of a subsistence agrarian economy such as this meant that a considerable portion of the population lived in relative poverty. This is evident in hearth tax assessments for Montgomeryshire from the late seventeenth century, which show that as many as 57% of households were deemed too poor to pay the tax.^97^ Industrial developments began to expand in south Wales in areas such as Merthyr in the 1760s, but similar developments did not take place in Montgomeryshire and Radnorshire.^98^ Although lead mining was a feature in Montgomeryshire near Llanidloes and Llangynog, mines and smelteries declined in the early eighteenth century and were not an important feature of the economic landscape of the region.^99^ Montgomeryshire was predominantly agricultural, but parts of the county were also heavily involved in the woollen trade throughout the eighteenth century. However, the woollen trade in the county remained a scattered, domestic industry dominated by the Shrewsbury Drapers Company, and only gradually became mechanized from the 1780s.^100^ Even the poorest cottages in Montgomeryshire could probably afford a spinning wheel, and many would have been involved in carding and weaving, which could have helped to supplement incomes.^101^ The economy in Radnorshire was also predominately agricultural, but unlike Montgomeryshire, was not actively involved in the woollen trade. The bulk of the population would have been poor peasants.^102^

The key characteristics which have emerged thus far are the relatively harsh economic circumstances across the region studied, with some pockets of wool production which may have improved circumstances for the poorest in those regions, and the presence of established courtship customs which increasingly allowed for, and probably involved, sexual contact. Given these circumstances, Adrian Wilson’s analysis of illegitimacy in eighteenth-century London proves particularly useful. Wilson found that, in general, the number of children admitted to the Foundling Hospital corresponded strongly to changes in grain prices.^103^ Wilson argued that most foundlings were illegitimate, and their conceptions resulted from a particular model of courtship which existed in the capital, but not in rural areas.^104^ In this model, marriages in London were ‘courtship-led’, meaning courting couples engaged in sex before a promise to marry had been made, and committed to marriage only after conception had occurred.^105^ The inverse of this, which Wilson argues prevailed elsewhere in rural areas of pre-industrial England, was courtship that was ‘marriage-led’, meaning a decision to marry preceded sexual contact.^106^ Marriage in both models was still very much contingent on economic circumstances. However, in the marriage-led model, sexual activity did not take place until after the couple was financially able to marry and set up a household. The combination of a courtship-led model of marriage and economic instability meant that many unmarried couples in London ended up conceiving but not marrying because of their financial circumstances, thus resulting in the birth of more illegitimate children.

The evidence from Montgomeryshire and Radnorshire points to a similar courtship-led model of marriage within the context of poor economic circumstances. Evidence from both counties supports this, as both Montgomeryshire and Radnorshire experienced deteriorating economic conditions which corresponded to increases in the illegitimacy ratio. Radnorshire experienced the cumulative effects of harsh weather and crop failure between 1727 and 1730, which saw the poor rate double, and in the following decade death rates increased substantially.^107^ The 1760s also saw periods of hardship, reflected in increased demand on relief from poor law officials, and the 1770s experienced further deficient harvests and considerable increases in poor rates.^108^ These decades roughly correspond with the more marked increases in the illegitimacy ratio in Radnorshire found in Figure 3. The early peak in illegitimacy in Montgomeryshire between 1700 and 1709 corresponds with a series of poor harvests due to long winters and cold summers which resulted in famine, and which probably also affected conditions in neighbouring Radnorshire.^109^ Three years during this decade also experienced ‘crisis mortality’ when the number of burials surpassed the number of baptisms by at least 10%. In 1709, crisis mortality in Montgomeryshire reached 20%, and in 1700 and 1708 this rose to over 30%.^110^ The plateau in illegitimacy between 1710 and 1749 roughly corresponds with a similar plateau in the number of poor law claimants throughout the county, which suggests a period of relative economic stability.^111^ However, the rise in illegitimacy from 1740 onwards is not matched by a rise in poor law claimants until midway through the 1760s.^112^ Further research into the correlation between economic circumstances and illegitimacy levels in Wales during the eighteenth century is needed, but preliminary analysis would suggest that economic circumstances were a significant contributing factor to increases in childbirths outside of wedlock.

Additional links between economic circumstances, illegitimacy and marriage can be found in the areas where the woollen trade was clearly active. Women would have been employed at the domestic level in activities such as combing, carding and spinning.^113^ In a study of marriage patterns, Bridget Hill examined evidence from the wool producing parish of Culcheth, Lancashire, where by the 1760s the illegitimacy ratio had reached 8.1%, and it continued to increase, reaching 10% by the early nineteenth century. In some years, illegitimacy could be as high as 30% of all baptisms.^114^ The majority of these women were handloom weavers who, Hill argues, made a conscious decision to separate childbearing from marriage by remaining in their parents’ home after the birth of their illegitimate children. This decision was beneficial for both the mothers and their families as it was less expensive than establishing a new home within marriage, and it allowed the young woman’s parents to retain her labour within the home. Griffin also found evidence to suggest that increased employment opportunities for women could have provided the mothers of illegitimate children with the means to support themselves and their children without having to marry.^115^ Although less technologically advanced, the woollen trade in Montgomeryshire could possibly have provided similar opportunities for unmarried mothers. However, this appears to have not been the case. Not only did the wool-producing county have lower levels of illegitimacy than Radnorshire overall, but parishes where the woollen trade was established, such as Meifod and Machynlleth, had some of the lowest levels of illegitimacy.^116^ The illegitimacy ratio in both parishes in the 1790s was 4.4%, which was considerably lower than the 7.5% county aggregate, and slightly lower than the national average 5.1% in England.^117^ This evidence would suggest that when economic circumstances allowed, the courtship-led model of marriage in Wales could, and frequently did, lead to marriage rather than illegitimacy.

Regional sensitivities to economic circumstances may also account for the disparity in the frequency with which fathers in Montgomeryshire and Radnorshire were identified. In addition to having relatively low levels of illegitimacy, the parishes of Meifod and Machynlleth reported some of the highest levels of identified paternity, with 80 and 100% in the 1790s respectively. As previously noted, some historians have suggested that the frequent identification of fathers may indicate community awareness, and even acceptance, of the relationship between courting couples. Caution must be taken with this assumption, though, because these identified fathers included men who had committed rape, incest and adultery, or had exploited their positions of power to take advantage of women subordinate to them.^118^ Therefore, not all identified fathers can be interpreted as suitors. An alternative hypothesis may be that the identification of fathers represents a more-established means of formal affiliation, which existed for financial reasons. Poor law legislation had held both parents financially accountable for the support of their children since the sixteenth century, but because mothers were often sole carers, the financial burden most often fell to fathers.^119^ An Act of 1733 established the formal legal mechanism that would, in theory, ensure fathers would indemnify the parish against the cost of supporting illegitimate children.^120^ The more frequent recording of fathers in Montgomeryshire could represent one component of the affiliation system as it operated there, which may have been absent in Radnorshire.

A further indication that this may be the case can be found the responses to Rural and Town Queries. Parish officials were asked three questions relating to illegitimacy, one of which was:

What number of bastards have been chargeable to your parish? And what has been the expense occasioned by them during each of the last five years? And how much of the expense has been recovered from the putative father? And how much from the mothers?^121^

Ten Montgomeryshire parishes and two Radnorshire parishes returned responses to this question.^122^ Most reported having spent between £100 and £1,000 on the maintenance of illegitimate children over the preceding five-year period, and most recovered between £50 and £230 from fathers. Only five parishes reported recovering any costs from mothers, which amounted to no more than a few pounds. Although the limited number of responses do not allow for quantitative analysis similar to Thomas Nutt’s study of paternal financial responsibility under the old poor law, some inferences can be made.^123^ Most parishes appear to have recouped less than one-third of what they spent on the maintenance of illegitimate children, which is considerably less than the amounts recouped in England.^124^ This is likely to be indicative of the persistent poverty experienced to different extents across both counties. What is perhaps most telling is the scarcity of answers to this question from Radnorshire. The parish of Clyro was the only one to provide amounts for actual annual expenditure and costs recovered, which were comparable to Montgomeryshire.^125^ However, the response from the parish of Old Radnor may be more telling. Officials there reported paying 2s per week per chargeable illegitimate child, with fathers contributing an additional 1s. The reason given for the practice was that ‘if more were demanded it would force the father on the parish’.^126^ Although far more research is required, this response presents the intriguing possibility that endemic poverty throughout the county may have rendered futile any formal affiliation processes, which is also reflected in patterns of paternal identification at the time of baptism. The high levels of illegitimacy and low levels of identification experienced in Radnorshire therefore demonstrate just how closely illegitimacy and economic circumstances were linked, especially when the marriage process was courtship-led.

## Conclusion

Many different sexual encounters can result in an illegitimate birth, but it is likely that most illegitimate children born in Wales during the eighteenth century were conceived in courtship. Although manifested in different ways, and in significantly different measure, patterns of illegitimacy across Wales were governed by a combination of cultural and economic factors surprisingly similar to those found in eighteenth-century London. Courtship-led marriage processes, which included practices such as bundling, were most likely a significant contributing factor to the increased levels of illegitimacy seen across mid-Wales during the century. The sexual experiences of Welsh courting couples changed over this period, but not because of a sexual revolution led by profound shifts in sexual knowledge. Rather, the changes can be ascribed to a decline in the traditional modes of regulation enforced by the ecclesiastical courts. Central to our understanding of why these changes in sexual practice had such a considerable impact on levels of illegitimacy are the specific regional economic circumstances in which these courtship practices took place. These couples probably engaged in penetrative sex with the intention of marrying should conception occur, but once faced with that reality, their personal circumstances prevented them from doing so. In Wales, patterns of illegitimacy reveal the realities of life in a predominantly agrarian economy which existed, to varying degrees, on the edge of subsistence. Patterns of illegitimacy are therefore an important marker of the ways in which the broad changes which occurred during the eighteenth century were experienced at the local level.

Regional variations in levels of illegitimacy across Britain make it abundantly clear that there is no satisfactory explanation which addresses all circumstances in all locations. The experience of illegitimacy in mid-Wales is only one manifestation of the dynamic interplay between sexual practice and socioeconomic reality. Although these are the most apparent, and therefore most useful for this preliminary study of Welsh evidence, they are not the only variables which need to be considered. Other promising fields of enquiry include the role of migration from mid-Wales to the industrializing south and elsewhere, more detailed analysis of poor law rates and expenditure, the socioeconomic background of parents, maternal and paternal responsibility and the analysis of subsequent marriages or the bearing of additional illegitimate children. The experiences of bearing, and being, an illegitimate child, including the presence or absence of stigmatization, maternal survival strategies and the provision of care to unmarried mothers also warrants further inquiry.^127^ Marriage carried no guarantee of economic stability, but it did have the power to legitimize children.^128^ That an ever-increasing number of Welsh couples did not end up marrying may indicate the diminishing importance. Social attitudes towards illegitimacy were complex and varied, and the ways in which they responded to a rising number of illegitimate children being born should be explored.^129^

Perhaps most importantly, the structural inequalities which existed in the eighteenth century that disadvantaged women, and unmarried women in particular, need to be considered more fully. This context is arguably where the direction of future studies of illegitimacy in Wales and in England should go, because ultimately women do not create illegitimate children; societies do. There are many other types of experiences beyond courtship which resulted in the birth of an illegitimate child, which also warrant further study. In effect, the study of illegitimacy may be better approached as a study of illegitimacies. This preliminary analysis has explored only a few facets of the evidence available for Wales, but Welsh records also reveal a diverse tapestry of sexual experience resulting in myriad forms of illegitimacy that cannot be attributed to a single pattern of sexual behaviour, and cannot be quantified and mapped as unified demographic phenomena. Seeking a leading cause is relevant, particularly in an initial analysis such as this, but the diversity of experiences should also be considered. The experience of illegitimacy in Wales in the eighteenth century was clearly unique in many ways, but it was also part of the broader British context, and needs to be considered as such.

## Disclosure statement

I have no financial interest or benefit arising from the direct applications of this research.

## Funding

This work was supported by Wellcome Trust [grant no. WT104885MA]; Social Sciences and Humanities Research Council of Canada [grant no. 752-2015-0033].

## Footnotes

^1^ For example, see A. Blaikie, *Illegitimacy, Sex and Society: north*-*east Scotland, 1750*–*1900* (Oxford, 1993); L. Leneman and R. Mitchison, *Sexuality and Social Control: Scotland 1660*–*1780* (Oxford, 1989).

^2^ P. Laslett, *Family Life and Illicit Love in Earlier Generations: essays in historical sociology* (Cambridge, 1977); P. Laslett, K. Oosterveen and R.M. Smith (eds), *Bastardy and its Comparative History* (London, 1980); R. Adair, *Courtship, Illegitimacy and Marriage in Early Modern England* (Manchester, 1996).

^3^ Laslett, *‘*Introduction’*, Bastardy, op. cit.,* 14, Adair, *op. cit.*, 50.

^4^ Laslett, ‘Introduction’, *Bastardy, op. cit.*, 30; Adair, *op. cit.* 52–64; E. Griffin, ‘Sex, illegitimacy and social change in industrializing Britain’, *Social History*, 38, 2 (2013), 139–61; A. Wilson, ‘Illegitimacy and its implications in mid-18th century London: the evidence of the Foundling Hospital’, *Continuity and Change*, 4, 1 (1989), 103–64.

^5^ Laslett, *Family Life*, *op. cit.*, 105–107; Laslett, ‘Introduction’, *Bastardy, op. cit.*, 3; Adair, *op. cit*., 15–16, 79; J.R. Gillis, *For Better, For Worse: British marriages, 1600 to the present* (Oxford, 1985), 128, 219; D. Levine and K. Wrightson, *The Making of an Industrial Society: Whickham, 1560*–*1765* (Oxford: 1991) as cited in R. Probert, ‘Introduction’ in R. Probert (ed.), *Cohabitation and Non*-*Marital Births in England and Wales, 1600*–*2012* (London, 2014).

^6^ T. Hitchcock, ‘Redefining sex in eighteenth-century England’, *History Workshop Journal*, 41 (1996) 72–90; R. Trumbach, *Sex and the Gender Revolution, vol 1: heterosexuality and the third gender in Enlightenment London* (Chicago, 1998); T. Laqueur, ‘Sex and desire in the Industrial Revolution’, in P. O’Brien and R. Quinault (eds), *The Industrial Revolution in British Society* (Cambridge, 1993), 100–23; F. Dabhoiwala, *The Origins of Sex: a history of the first sexual revolution* (London, 2012).

^7^ Laslett, ‘Introduction’, *Bastardy, op. cit.*; Adair, *op. cit.*; T. Nutt, ‘Illegitimacy, paternal financial responsibility, and the 1834 Poor Law Commission Report: the myth of the old poor law and the making of the new’, *Economic History Review*, 63, 2 (2010), 355–61; N. Rogers, ‘Carnal knowledge: illegitimacy in eighteenth-century Westminster’, *Journal of Social History*, 23, 2 (1989), 355–75.

^8^ Wilson,‘Illegitimacy’, *op. cit.*, 134; Rogers, *op. cit.*, 369; Laslett, ‘Introduction’, *Bastardy, op. cit.*, 53–61.

^9^ Rogers, *op. cit.*, 39; Laslett, ‘Introduction’, *Bastardy, op. cit.*, 53–61.

^10^ Griffin, *op. cit.*, 143.

^11^ Griffin, *op. cit*.; Wilson, ‘Illegitimacy’, *op. cit.*

^12^ A. Brueton, ‘Illegitimacy in South Wales 1660–1870’ (Ph.D., Leicester, 2014); A. Brueton, ‘Courtship, illegitimacy and marriage in a rural community: the Upper Tywi Valley, 1760–1860’ (M.A., Trinity University College Carmarthen, 2007); A. Muir, ‘Illegitimacy in eighteenth-century Wales: paternity, courtship, marriage and illicit sex’ (M.A., Swansea University, 2011); A. Muir, ‘Illegitimacy in eighteenth-century Wales’, *Welsh History Review*, 26, 3 (2013), 351–88.

^13^ British Parliamentary Papers (BPP), *2nd Annual Report of the Poor Law Commissioners for England and Wales*, 1836, XXIX, Appendix D, 567–68.

^14^ Montgomery (parish register transcripts published by the Montgomeryshire Genealogical Society unless otherwise stated): Caereinion (hundred): Castell Caereinion (MR/C/15; MR/PR/03; MR/PR/04), Garthbeibio (MR/PR/25; MR/PR/26), Llanfair Caereinion (MR/PR/65; MR/PR/66; MR/PR/67; MR/PR/68), Llangadfan (MR/PR/37; MR/PR/38; MR/PR/39), Llangyniew (MR/C/21; MR/PR/18; MR/PR/19; MR/PR/20); Cefeiliog (hundred): Cemmaes (MR/C/39; MR/PR/99), Darowen (MR/C/40; MR/PR/102; MR/PR/103), Llanwrin (MR/C/38; MR/PR/95), Machynlleth (MR/C/36; MR/C/37; MR/PR/88; MR/PR/89; MR/PR/90); Llanfyllin (hundred): Llanfihangel-yng-Ngwynfa (MR/C/30; MR/PR/54), Llanfyllin (MR/C/24; MR/PR/56; MR/PR/57; MR/PR/58; MR/PR/59), Llanrhaeadr ym Mochnant (Clwyd Family History Society, vols 1–4), Llanwddyn (MR/PR/50; MR/PR/51), Meifod (MR/C/16; MR/C/17; MR/PR/05; MR/PR/06; MR/PR/07); Llanidloes (hundred): Carno (MR/PR/114; MR/PR/115; MR/PR/116), Llandinam (MR/C/48; MR/C/49; MR/PR/107; MR/PR/108), Llanwnog (MR/C/55; MR/PR/141; MR/PR/142), Trefeglwys (MR/C/43; MR/PR/103; MR/PR/104); Newtown (hundred): Aberhafesp (MR/PR/80; MR/PR/81), Berriew (MR/C/27; MR/PR/42; MR/PR/43; MR/PR/44; MR/PR/45), Betws Cedewain (MR/PR/85; MR/PR/86; MR/PR/87), Newtown (MR/C/23; MR/PR/28; MR/PR/29); Ystrad Marchell (hundred): Buttington (MR/PR/92; MR/PR/93; MR/PR/94), Guilsfield (MR/C/32; MR/PR/30; MR/PR/31; MR/PR/32; MR/PR/33; MR/PR/34; MR/PR/35), Llandrinio (MR/PR/61; MR/PR/62), Welshpool (MR/C/08; MR/C/09; MR/C/10; MR/C/11). Radnorshire (Transcripts published by the Powys Family History Society): Cefnllys/Knighton (hundreds): Beguildy (POW/PR10aCD), Llanbadarn Fawr (POW/PR08CD), Llanbadarn Fynydd (POW/PR09CD), Llanbister (POW/PR07CD); Radnor (hundred): Gladestry (POW/PR17CD), Glascwm (POW/PR01CD), Llanfihangel Nant Melan (POW/PR15CD), Llansantffraed-in-Elwel (POW/PR02CD), New Radnor (POW/PR16CD), Norton (POW/PR06CD).

^15^ Laslett, *Family Life*, *op. cit.*, 120.

^16^ Adair, *op. cit.*, 32 chose to include illegitimate children found in burial registers in his analysis.

^17^ L. Stone, ‘Kinship and forced marriage in early eighteenth-century Wales’, *Welsh History Review*, 17 (1995), 356; M. Anderson (ed.), *British Population History: from the Black Death to the present day* (Cambridge, 1996), 113.

^18^ Laslett, ‘Introduction’, *Bastardy, op. cit.*, 14.

^19^ Adair, *op. cit*., 63.

^20^ Adair, *op. cit.*, 51–62 (Regions are as follows: west includes Derbyshire, Gloucestershire, Herefordshire, Monmouthshire, Shropshire, Staffordshire, Warwickshire, Worcestershire; northwest includes Cheshire, Cumberland, Lancashire, Westmorland).

^21^ English parishes: Herefordshire (Transcripts held by Hereford Archives & Records Centre): Brilley (AK37), Kington (AP68), Little Dewchurch (open shelves); Shropshire (Transcripts held by Shropshire Archives): Bishops Castle (open shelves), Cardeston (ANG/ARA/615), Hordley (ANG/ARA/471), Kinnerley (open shelves), Ludlow (open shelves), Munslow (open shelves), Onibury (ANG/ARA/373), Oswestry (ANG/ARA/398), and Uffington (ANG/ARA/397).

^22^ J.G. Jenkins, ‘The welsh woollen industry’ in D. Moore (ed.), *Wales in the Eighteenth Century* (Swansea, 1976), 95.

^23^ K. Oosterveen, R.M. Smith and S. Stewart, ‘Family reconstitution and the study of bastardy’ in Laslett, Oosterveen and Smith (eds), *op. cit.*, 104; Adair, *op. cit*., 78–79.

^24^ B. Reay, ‘Sexuality in nineteenth-century England: the social context of illegitimacy in rural Kent’, *Rural History*, 1 (1990), 221.

^25^ Laslett, *Family Life*, *op. cit.*, 109–110.

^26^ Reay, *op. cit*., 221.

^27^
*ibid.*

^28^ R.E. Jones, ‘Infant mortality in rural north Shropshire, 1561–1810’, *Population Studies*, 30, 2 (1976), 305–17, 313.

^29^ Laslett, *Family Life*, *op. cit.*, 105–107; Laslett, ‘Introduction’, *Bastardy, op. cit.*, 3, 20–22; Adair, *op. cit.*, 15–16, 64–65, 79.

^30^ L. Gowing, ‘Ordering the body: illegitimacy and female authority in seventeenth-century England’ in M.J. Braddick and J. Walter (eds), *Negotiating Power in Early Modern Society: order, hierarchy and subordination in Britain and Ireland* (Cambridge, 2001), 45; A. Wilson, *Ritual and Conflict: the social relations of childbirth in early modern England* (Farnham, 2013), 8.

^31^ Reay, *op. cit*., 237.

^32^ Griffin, *op. cit.*

^33^ See also E. Hurren and S. King, ‘Courtship at the corner’s court’, *Social History*, 40 (2015), 185–207.

^34^ National Library of Wales *Gaol Files* (subsequently NLW): 4/523/4 (Margaret Matthews, 1756); 4/53/3 (Mary Jones, 1762); 4/188/1 (Elizabeth Evans, 1764); 4/388/5 (Margaret Thomas, 1788); 4/390/4 (Ann Watkin, 1797).

^35^ NLW 4/188/1.

^36^ NLW 4/388/5.

^37^ NLW 4/523/4.

^38^ NLW 4/188/1.

^39^ Griffin, *op. cit.*, 149.

^40^ C. Stevens, *Welsh Courting Customs* (Llandysul, 1993), 104.

^41^
*ibid.*, 83–87.

^42^
*ibid*., 82; Laslett, ‘Introduction’, *Bastardy, op. cit.*, 8.

^43^ Muir, ‘Illegitimacy’, *op. cit.*, 370; Adair, *op. cit.*, 6.

^44^ Stevens, *op. cit.*, 86–87.

^45^ Mr Pratt (Samuel Jackson), *Gleanings through Wales, Holland and Westphalia* (London, 1797), 105.

^46^ BPP, *Royal Commission of Inquiry into Administration and Practical Operation of Poor Laws, Answers to Town Queries*, 1834, XXXVI (Town Queries), 180a.

^47^
*ibid.*

^48^ NLW 4/760/4 (Elizabeth Jones, 1816).

^49^ NLW 4/259/5 (Griffith Roberts, 1824).

^50^ Hitchcock, *op. cit.*; Dabhoiwala, *op. cit.*; Laqueur, *op. cit.*; S. Toulalan, *Imagining Sex, Pornography and Bodies in Seventeenth*-*Century England* (Oxford, 1997).

^51^ Hitchcock, *op. cit.*, 76.

^52^ Hitchcock, *op. cit.*, 78; Dabhoiwala, *op. cit*., 141–42.

^53^ Hitchcock, *op. cit.*, 79–80.

^54^
*ibid.*, 81.

^55^
*ibid.*, 82–84.

^56^
*ibid.*, 83.

^57^
*ibid.*, 82.

^58^
*ibid.*, 83.

^59^ G.H. Jenkins, *The Foundations of Modern Wales, 1642*–*1780* (Oxford, 1993), 398.

^60^ E. Rees, ‘The development of the book trade in eighteenth-century Wales’, *Library*, 24 (1969), 34.

^61^
*ibid.* 39.

^62^ E. Rees, *Libri Walliae: a catalogue of Welsh books and books printed in Wales 1546*–*1820* (Aberystwyth, 1987).

^63^ E.M. White, *The Welsh Bible: a history* (Stroud, 2007), 62. Other works by Woodward which were translated by the SPCK into Welsh include *Cyngor yr eglwyswr: i ŵr ieuangc newydd dderbyn conffirmasiwn gan yr Esgob. Neu'r hyn a elwir yn gyffredinol, Bedydd*-*Esgob* (*Council of the Churchman: A Young Man Receiving Confirmation from the New Bishop* (1703) and *Llaw*-*lyfr y Gwir Gristion* (*Handbook of the True Christian)* (1716).

^64^ A. Withey, *Physick and the Family: health, medicine and care in Wales, 1600*–*1750* (Manchester, 2011), 58–63.

^65^ Toulalan, *op. cit.*

^66^ Dabhoiwala, *op. cit.*, 71–78.

^67^ R.B. Outhwaite, *The Rise and Fall of English Ecclesiastical Courts 1550*–*1860* (Cambridge, 2007), 78–94.

^68^
*ibid*., 83.

^69^ A. Clark, ‘Heterosexuality: Europe and North America’ in J. Peakman (ed.), *A Cultural History of Sexuality in the Enlightenment* (Oxford, 2011), 43; Outhwaite, *op. cit.*, 84.

^70^ Dabhoiwala, *op. cit.*, 71–77.

^71^ Jenkins, *The Foundations*, *op. cit*., 347, 356, 367.

^72^
*ibid*., 376.

^73^ D.W. Howell, *The Rural Poor in Eighteenth*-*Century Wales* (Cardiff, 2000), 15; M. Humphreys, *The Crisis of Community Montgomeryshire, 1680*–*1815* (Cardiff, 1996), 176.

^74^ Griffin, *op. cit*., 154.

^75^ Gowing, ‘Ordering the body’, *op. cit.*; L. Gowing, ‘Knowledge and experience, c. 1500–1750’ in S. Toulalan and K. Fisher (eds) *The Routledge History of Sex and the Body: 1500 to the present* (London, 2013), 242.

^76^ Nutt, *op. cit.*

^77^ Griffin, *op. cit.*, 146.

^78^ Laslett, ‘Introduction’, *Bastardy, op. cit.*, 54–55.

^79^ D. Levine, *Family Formation in an Age of Nascent Capitalism*, ch. 9, cited in Leneman and Mitchison, *op. cit*., 5.

^80^ Griffin, *op. cit*., 143.

^81^ Wilson, ‘Illegitimacy’, *op. cit.*

^82^ E. Shorter, ‘Illegitimacy, sexual revolution and social change in modern Europe’, *Journal of Interdisciplinary History*, 2 (1971), 237–72.

^83^ L.A. Tilley, J.W. Scott and M. Cohen, ‘Women’s work and European fertility patterns’, *Journal of Interdisciplinary History*, 6 (1976), 447–476.

^84^
*ibid*., 470.

^85^
*ibid*., 472–76.

^86^ N. Goose, ‘How saucy did it make the poor? The straw plait and hat trades, illegitimate fertility and the family in nineteenth-century Hertfordshire’, *History*, 91, 404 (2006), 530–56.

^87^ Griffin, *op. cit*., 156–57.

^88^ Reay, *op. cit.*; S. King, ‘The bastardy-prone sub-society again: bastards and their fathers and mothers in Lancashire, Wiltshire, and Somerset’, in A. Levene, S. Williams and T. Nutt, (eds), *Illegitimacy in Britain, 1700–1920* (Basingstoke, 2005); Laslett, ‘Introduction’, *Bastardy, op. cit.*

^89^ Reay, *op. cit*.; Adair, *op. cit.*; K. Wrightson and D. Levine, ‘The social context of illegitimacy in early modern England’ in Laslett, Oosterveen and Smith (eds), *op. cit*., 158–75.

^90^ Humphreys, *op. cit*., 3, 39.

^91^ P.D.G. Thomas Moore, ‘Society, government and politics’ in Moore, *Wales in the Eighteenth Century*, *op. cit.*, 9–10. Population estimates for the years prior to 1801 are not available.

^92^ Jenkins, *Foundations*, *op. cit.*, 88; G.H. Jenkins, *A Concise History of Wales* (Cambridge, 2007), 36.

^93^ Howell, *op. cit*., 27; Humphreys, *op. cit*., 25; Jenkins, *Foundations*, *op. cit*., 87; N. Carslile, *A Topographical Diction of the Dominion of Wales* (London, 1811).

^94^ N. Ward, *A Trip to North*-*Wales* (London, 1701) in Jenkins, *Foundations*, *op. cit*., 87.

^95^ Humphreys, *op. cit*., 3, 16–20; Jenkins, *A Concise History*, *op. cit*., 10–11.

^96^ Howell, *op. cit*., 103; Humphreys, *op. cit*., 35, 64.

^97^ Howell, *op. cit*., 95; Humphreys, *op. cit*., 91.

^98^ R.O. Roberts, ‘Industrial expansion in South Wales’, in Moore, *op. cit*., 109–26.

^99^ Humphreys, *op. cit.*, 23–24.

^100^ Jenkins, ‘The Welsh woollen industry’, *op. cit.*

^101^
*ibid.*, 97.

^102^ O.S. Ashton, ‘Eighteenth-century Radnorshire’, *Radnorshire Society Transactions*, 40 (1970), 40.

^103^ Wilson, ‘Illegitimacy’, *op. cit.*, 131.

^104^ Wilson’s argument that most foundlings were illegitimate has been challenged: see A. Levene, ‘The origins of the children of the London Foundling Hospital, 1741–1760: a reconsideration’, *Continuity and Change*, 18 (2003), 201–35.

^105^
*ibid*., 134–36.

^106^
*ibid.*, 136.

^107^ Ashton, *op. cit*., 51.

^108^
*ibid*., 52.

^109^ Humphreys, *op. cit*., 64.

^110^
*ibid*., 72–73.

^111^
*ibid*., 89.

^112^
*ibid*., 89.

^113^ Jenkins, *A Concise History*, *op. cit*., 13.

^114^ B. Hill, ‘The marriage age of women and the demographers’, *History Workshop Journal*, 28 (1989), 143.

^115^ Griffin, *op. cit*., 157–58.

^116^ Jenkins, ‘The Welsh woollen industry’, *op. cit*., 99.

^117^ In Meifod, 20 of the 453 baptisms recorded in this decade were illegitimate, 16 of which named a putative father. In Machynlleth, 25 of the 573 baptisms in this decade were illegitimate, all of which listed a putative father: Laslett, ‘Introduction’, *Bastardy, op. cit.*, 14.

^118^ Evidence of this is limited, but can be found in baptism registers and other parish documents including bastardy bonds. For example, the 1743 baptism register for Llansilin, Denbighshire, includes an entry for a child named Richard Morris, born to Matthew Morris and his daughter Elizabeth, ‘by incest’ (Clwyd Family History Society, Llansilin parish register transcriptions, vol 2).

^119^ King, *op. cit*., 103.

^120^ King, *op. cit*., 103, citing 6 George II c. 31, *An Act for the Relief of Parishes and other Places from such Charges as may arise from Bastard Children….*

^121^ BPP, *Town Queries*, Appendix B.2, part V, question 59.

^122^ BPP, *Town Queries*, 650e to 656e; 661e-662e.

^123^ Nutt, *op. cit.*

^124^
*ibid*., 346: northern parishes were far more successful than their southern counterparts in recouping bastardy cost from putative fathers (82.6% of bastardy costs vs 41.5%).

^125^ BPP, *Town Queries*, 661e.

^126^ BPP, *Town Queries*, 662e.

^127^ See T. Evans, *Unfortunate Objects: lone mothers in eighteenth*-*century London* (Basingstoke, 2005); S. Williams, ‘The experience of pregnancy and childbirth for unmarried mothers in London, 1760–1866’, *Women’s History Review*, 20, 1 (2011), 67–86.

^128^ Evans, *op. cit.,* 51.

^129^ Reay, *op. cit*., 243.

